# Autophagy Induced by Simian Retrovirus Infection Controls Viral Replication and Apoptosis of Jurkat T Lymphocytes

**DOI:** 10.3390/v12040381

**Published:** 2020-03-31

**Authors:** Jingting Zhu, Lingyan Yang, Qibo Zhang, Jia Meng, Zhi-Liang Lu, Rong Rong

**Affiliations:** 1 Department of Biological Sciences, Xi’an Jiaotong-Liverpool University, 111 Ren’ai Road, Suzhou Dushu Lake Science and Education Innovation District, Suzhou Industrial Park, Suzhou 215123, China; zpzjt822@163.com (J.Z.); Jia.Meng@xjtlu.edu.cn (J.M.); Zhiliang.Lu@xjtlu.edu.cn (Z.-L.L.); 2 Department of Clinical Infection, Microbiology and Immunology, Institute of Infection and Global Health, University of Liverpool, Liverpool L69 7BE, UK; Qibo.Zhang@liverpool.ac.uk; 3 VRL-China, Suzhou 215123, China; lingyan.yang@vrlasia.cn; 4 Institute of Integrative Biology, University of Liverpool, Liverpool L69 7ZB, UK

**Keywords:** SRV, autophagy, apoptosis, autophagosome, viral replication

## Abstract

Autophagy and apoptosis are two important evolutionarily conserved host defense mechanisms against viral invasion and pathogenesis. However, the association between the two pathways during the viral infection of T lymphocytes remains to be elucidated. Simian type D retrovirus (SRV) is an etiological agent of fatal simian acquired immunodeficiency syndrome (SAIDS), which can display disease features that are similar to acquired immunodeficiency syndrome in humans. In this study, we demonstrate that infection with SRV-8, a newly isolated subtype of SRV, triggered both autophagic and apoptotic pathways in Jurkat T lymphocytes. Following infection with SRV-8, the autophagic proteins LC3 and p62/SQSTM1 interacted with procaspase-8, which might be responsible for the activation of the caspase-8/-3 cascade and apoptosis in SRV-8-infected Jurkat cells. Our findings indicate that autophagic responses to SRV infection of T lymphocytes promote the apoptosis of T lymphocytes, which, in turn, might be a potential pathogenetic mechanism for the loss of T lymphocytes during SRV infection.

## 1. Introduction

Macroautophagy, hereafter referred as autophagy, is an evolutionarily conserved degradation process that mediates cellular homeostasis under a variety of stressful conditions, including viral invasion [1,2,3]. Studies have demonstrated that viral infection could either activate or inhibit the autophagic pathways, which might, in turn, affect viral replication and pathogenesis [4,5,6,7,8]. As an intrinsic cellular response, autophagy can directly eliminate an invading pathogen, such as DNA viruses (herpes simplex virus 1) or RNA viruses (Sindbis and Japanese encephalitis viruses), by delivering it for lysosomal degradation [9,10,11]. In addition, autophagy could modulate innate and adaptive immunity to combat viral infection [12,13]. On the other hand, some viruses have developed strategies to escape, or even subvert, this pathway, for their own benefit [14,15,16]. For example, matrix protein 2 of influenza virus has been shown to block the degradation of autophagosome, which could serve as a scaffold to support viral replication [16,17,18,19]. The interplay between autophagy and viral replication in the infected T cells has not been explored in the case of simian type D retrovirus (SRV) infection.

The autophagic pathway is commonly recognized as a cytoprotective mechanism to facilitate cell survival and to suppress cell death. In contrast, excessive or persistent autophagy has been shown to promote cell death, directly or indirectly, through caspase-dependent apoptosis [20,21]. Autophagy has also been proposed to play either pro-survival or pro-death roles in lymphocytes [22,23,24]. Autophagy and apoptosis both play major roles in determining cell fate, which overlap with respect to the response to viral infection [25,26,27]. The interplay between autophagy and apoptosis is complex and mainly regulated by the proteins with dual roles in the two pathways [28,29]. Recent studies have demonstrated that the autophagosomal membrane forms a platform for the formation of an intracellular death-inducing signaling complex (iDISC), which could recruit and mediate the activation of caspase-8 and, thus, promote apoptosis [30,31,32]. However, the functional association between autophagy and cell viability during SRV infection of T lymphocytes has not been investigated. 

SRV is an enveloped, single-stranded RNA virus that belongs to the genus of *Beta-retrovirus* [33,34]. SRV mainly infects Asian macaques and it is an etiologic agent of spontaneous simian immunodeficiency syndrome (SAIDS). Pathologic manifestations of SRV infection vary from lymphadenopathy and hematologic abnormality to severe immune deficiency [35,36,37,38]. SRV exhibits a broad tropism for cells of both the lymphoid and non-lymphoid tissues, and the depletion of T lymphocytes is commonly detected in infected monkeys during the late stage of infection [39,40,41,42]. However, the effects of SRV infection on T cell biology are not well understood. SRV-8 is a subtype of SRV that was recently isolated from cynomolgus monkeys of Cambodian ancestry background [43], and it is closely related to SRV-4, which can cause severe and lethal disease in Japanese macaques (*Macaca fuscata*) [44]. Similar to other SRVs that can infect human T cell lines in vitro [40,45,46], SRV-8 can infect and replicate in Jurkat T lymphocytes [47]. Understanding the effect of SRV-8 infection on Jurkat T lymphocytes could enhance our knowledge in order to understand the mechanism of T lymphocyte depletion that is induced by retroviruses.

In this study, we investigated the effects of SRV-8 infection on the autophagic and apoptotic pathways of Jurkat T lymphocytes, as well as the potential role of autophagy in regulating viral replication and apoptosis. Our results reveal that SRV-8 infection could enhance the autophagic flux and increase apoptosis in infected Jurkat cells. Moreover, the inhibition of autophagy that is mediated by knockdown of Beclin1 was shown to increase SRV-8 replication in Jurkat cells. Additionally, the results suggest that the enhanced formation of autophagosomes might recruit procaspase-8 for its activation and, thus, lead to apoptosis of SRV-8-infected Jurkat cells.

## 2. Materials and Methods

### 2.1. Cell Lines, Virus Stock Preparation and Viral Infection

The Jurkat cell line (human T lymphocytes; ATCC^®^ TIB-152™, Manassas, VA, USA) and HEK293T cell line (human embryonic kidney cells; ATCC^®^ CRL-11268™) were kindly provided by Prof. Z. Lu, Xi’an Jiaotong-Liverpool University, China. The Raji cell line (human B lymphocytes) was purchased from the Chinese Academy of Sciences Cell Bank, Shanghai. Jurkat cells and Raji cells were grown in RPMI1640 (Hyclone, St Louis, MO, USA) complete medium supplemented with 5% (*v/v*) or 10% (*v/v*) FBS (Gibco, Waltham, USA), respectively. The HEK293T cells were grown in DMEM (Hyclone) complete medium supplemented with 10% (*v/v*) FBS. All complete media were also supplemented with 100 U/mL penicillin and 100 μg/mL streptomycin.

SRV-8 virus stock, SRV8/SUZ/2012 (GenBank accession number: KU605777), was kindly provided by VRLcn Ltd. (Suzhou, China) [43]. To propagate the infectious viral particles, 2 × 10^6^ Raji cells were co-cultured with 1 × 10^6^ SRV-8-infected Raji cells in the complete medium. When more than 70% of the cells displayed a cytopathic effect, the cells were pelleted, and the virus-containing supernatant was harvested and then filtered through the Nalgene™ Rapid-Flow™ 0.45 µm filter (#168-0045; Thermo Fisher Scientific, Waltham, MA, USA). The copy numbers of SRV genome RNA in the harvested supernatant were estimated while using qRT-PCR analysis.

For SRV-8 infection of Jurkat cells, 2 × 10^5^ Jurkat cells were cultured in 1 mL of the complete medium for 3 h, followed by incubation with known amounts of SRV-8 for 18 h at 37 °C. Approximately 1.5 × 10^8^ genome RNA copies on 10^5^ Jurkat cells corresponds to a multiplicity of infection (MOI) of 0.06. Equal amounts of Jurkat cells were incubated with the supernatant of uninfected Raji cells and served as the control uninfected cells. The cells were centrifuged, washed three times with PBS, resuspended, and maintained in the complete medium for the indicated time to remove the free viral particles after infection. 

### 2.2. Immunofluorescence Staining

The cells were seeded on 0.01% poly-L-lysine (#P4832; Sigma, St Louis, MO, USA)–coated Nunc™ Lab-Tek™ Chamber Slides (#177380PK; Thermo Fisher Scientific, Waltham, MA, USA), and fixed in 4% paraformaldehyde for 15 min. at room temperature. The cells were then permeabilized with pre-chilled pure methanol for 20 min. at −20 °C, followed by blocking with 5% BSA and 0.3% (*v/v*) Triton X-100 in PBS for 1 h at room temperature. The cells were then stained with the indicated primary antibody: anti-LC3B (1:200; #3836, Cell Signaling Technology, Danvers, MA, USA), anti-procaspase-8 (1:60; #746109, R&D Systems, USA), anti-SQSTM1/p62 (1:500; #ab91526, Abcam, Cambridge, UK) overnight at 4 °C. After incubation, the cells were washed three times with PBS and then incubated with the secondary antibody: FITC-conjugated swine anti-rabbit antibody (1:30; #F020502, DAKO, Japan) and/or Alexa Fluor^®^ 555-conjugated goat anti-mouse antibody (1:1000; #A21424, Invitrogen, Shanghai, China) for 2 h at room temperature. After rinsing three times with PBS, the cells were incubated with Hoechst (1:5000 dilution in PBS; #H1399, Thermo Fisher Scientific, Waltham, USA) for 10 min. at room temperature. The stained cells were then mounted with ProLong™ Gold Antifade Mountant (#P36934; Thermo Fisher Scientific, Waltham, USA) and then imaged with a ZEISS LSM 880 Confocal Laser Scanning Microscope.

### 2.3. Flow Cytometry Analysis

The apoptosis of Jurkat cells was analyzed by flow cytometry with Annexin V-PE/7-AAD staining. At the indicated time, cells were washed twice with cold PBS and stained using a PE Annexin V Apoptosis Detection Kit I (#559763; BD Biosciences, San Diego, CA, USA), according to the manufacturer’s instruction. For single Annexin V staining, cells were incubated with APC-conjugated Annexin V (#550474; BD Biosciences, San Diego, USA). After incubation, the apoptotic cells were detected using a FACSCalibur (BD Biosciences, San Diego, USA) instrument and the data were analyzed with Cell Quest Pro software. 

### 2.4. Western Blot Analysis and Co-Immunoprecipitation Assay

The cells were lysed in Laemmli Sample Buffer (24 mM Tris-HCI at pH 6.8, 0.8% SDS, 2% (*v/v*) beta-mercaptoethanol and 20% (*v/v*) glycerol) supplemented with EDTA-free complete™ protease inhibitor cocktail (#04693116001; Roche, Basel, Switzerland) on ice for 10 min. The nuclear contents in the cell lysates were sheared by sonication (Q700, Qsonica, Newtown, CT, USA) and whole lysates were heated at 100 °C for 10 min., being separated by electrophoresis on 10% or 12% SDS-PAGE gels, and transferred onto 0.2 μm PVDF membranes (Millipore, Burlington, MA, USA). Membranes were then blocked with 5% (*w/v*) skimmed milk in PBS-T (PBS containing 0.1% (*v/v*) Tween-20) and immunoblotted with the indicated antibodies. The protein bands were visualized and quantified using an Odyssey Infrared Imaging System (LI-COR, Lincoln, USA).

For co-immunoprecipitation, cells were lysed at 4 °C for 30 min. in lysis buffer (20 mM Tris-HCl at pH 7.6, 150 mM NaCl, 1% (*v/v*) NP-40 and 10% *(v/v*) glycerol) supplemented with EDTA-free complete™ protease inhibitor cocktail. The cell lysates were pre-cleaned by incubating with BSA-blocked protein A beads (#IPA300; Repligen, Waltham, MA, USA) for 30 min. at 4 °C, and then subjected to immunoprecipitation with an anti-LC3B antibody (#3836, Cell Signaling Technology, USA). After washing three times with lysis buffer, the beads were heated in Laemmli Sample Buffer for 10 min. at 95 °C and then subjected to western blot analysis. 

The primary antibodies used were: rabbit monoclonal anti-LC3B antibody (1:1000 dilution; #3836, Cell Signaling Technology, Danvers, MA, USA); rabbit monoclonal anti-cleaved caspase 3 antibody (1:1000 dilution; #9664, Cell Signaling Technology, USA); rabbit monoclonal anti-cleaved caspase 8 antibody (1:1000 dilution; #9496, Cell Signaling Technology, USA); rabbit polyclonal anti-cleaved caspase 9 antibody (1:1000 dilution; #9501, Cell Signaling Technology, USA); rabbit monoclonal anti-Beclin1 antibody (1:1000 dilution; #3495, Cell Signaling Technology, USA); rabbit monoclonal anti-ATG5 antibody (1:1000 dilution; #12994, Cell Signaling Technology, USA); mouse monoclonal anti-caspase 8 antibody (1:500 dilution; #66093-1-Ig, Proteintech Group, USA); and, mouse monoclonal anti-β-actin antibody (1:2000 dilution; #3700, Cell Signaling Technology, USA). The secondary antibodies were: IRDye^®^ goat anti-rabbit IgG (H + L) (#926-32211; LI-COR, Lincoln, USA) and IRDye^®^ donkey anti-mouse IgG (H + L) (#926-68072; LI-COR, Lincoln, USA).

### 2.5. RNA Interference and Lentiviral Transduction

RNA interference was used to knock down the expression of Beclin1 and ATG5 in Jurkat cells. The sequences for Beclin1 shRNA, ATG5 shRNA, and scrambled control shRNA are: 5′-TCCCGTGGAATGGAATGAGATT-3′, 5′-AGCAGAACCATACTATTTGCTT-3′, and 5′ATCTCGCTTGGG CGAGAGTAAG-3′, respectively. Each shRNA was subcloned into a Dharmacon™ TRIPZ™ lentiviral vector (pTRIPZ) and cotransfected into HEK293T cells, together with packaging plasmids using Fugene^®^ 6 Transfection Reagent (#E2691; Promega, Madison, WI, USA). The resulting supernatant containing shRNA-expressing lentivirus was used to transduce Jurkat cells. For transduction, the Jurkat cells were incubated with the lentiviral supernatants in the presence of polybrene (#H9268; Sigma, St Louis, USA) for 10 min. followed by spinoculation for 30 min. at 800× *g*. Puromycin was used to select the stably transduced Jurkat cell populations. To induce the expression of shRNA in stable Jurkat cells, 1 μg/mL of doxycycline hyclate (#ST039A; Beyotime, China) was used to treat the cells for the indicated time. The pTRIPZ vector and packaging plasmids were a gift from Dr. F. Kappes, Xi’an Jiaotong-Liverpool University, China.

### 2.6. Realtime Quantitative RT-PCR

Viral RNA was isolated from 140 µL sample of culture medium using a Viral RNA Extraction Kit (#DP316-R; TIANGEN, China). The isolated RNA was treated with RQ1 RNase-Free DNase (#M6101; Promega, Madison, USA) and then reversed transcribed into cDNA using Reverse Transcriptase M-MLV (RNase H-) Kit (#2641; TaKaRa, China), according to the manufacturer’s protocol. The primers and probe for SRV genome and the standard SRV plasmid were kindly provided by VRLcn Ltd. (Suzhou, China). The copy number of the viral genome in the cDNA was determined using Premix Ex Taq (Probe qPCR) Kit (#RR390A; TaKaRa, China) with realtime qPCR. 

The total RNA was isolated with TRI Reagent^®^ (#T9424; Sigma, St Louis, USA) followed by RNase-free DNase treatment and cDNA synthesis to examine mRNA levels of death receptors and ligands in cells, on day 10 post-infection, as described above. Realtime qPCR was performed using SYBR Premix Ex Taq (Tli RNase H Plus) Kit (#RR420; TaKaRa, China). GAPDH was used as an internal control for normalization. The primers were used as follows: Fas forward 5′-TGAAGGACATGGCTTAGAAGTG-3′ and reverse 5′-GGTGCAAGGGTCACAGTGTT-3′; FasL forward 5′-TGCCTCTGGAATGGGAAGAC-3′ and reverse 5′-TGCAAGATTGACCCCGGAAG-3′; TRAIL forward 5′-CGACTGCCTGGCTGACTTAC-3′ and reverse 5′-GATCACGATCAGCACGCAGG-3′; DR5 forward 5′-AAGGTGGCTAAAGCTGAGGC-3′ and reverse 5′-GCTTGGCAAGTCTCTCTCCC-3′. The primers of GAPDH were kindly provided by VRLcn Ltd. (Suzhou, China). All of the realtime qPCR reactions were run on the 7500 Fast Real-Time PCR System (Applied Biosystems, Waltham, MA, USA).

### 2.7. MTT Colorimetric Assay

The cell viability was examined using the MTT colorimetric assay. After the indicated treatment, 100 µL of 5mg/mL MTT solution (#M2818, Sigma, St Louis, MO, USA) was added into 1 mL cell suspension in a 24-well plate. After 4 h of incubation at 37 °C and 5% CO_2_, the cells were pelleted and resuspended in 500 µL of DMSO. Absorbance at 570 nm was read on a microplate reader (BioTek, Winooski, VT, USA). 

### 2.8. Statistical Analysis

All of the experiments were independently repeated for at least three times. All data were analyzed using the GraphPad Prism 6.0 (GraphPad Software, San Diego, USA) and presented as mean ± standard deviation (SD). Significant differences between two groups were analyzed while using unpaired Student’s *t*-test. Wherever indicated, the *p* values are as follows: *p < 0.05, **p < 0.01, ***p < 0.001.

## 3. Results

### 3.1. SRV-8 Infection Enhances Autophagosome Formation and Autophagic Flux in Jurkat Cells

**Figure 1:**
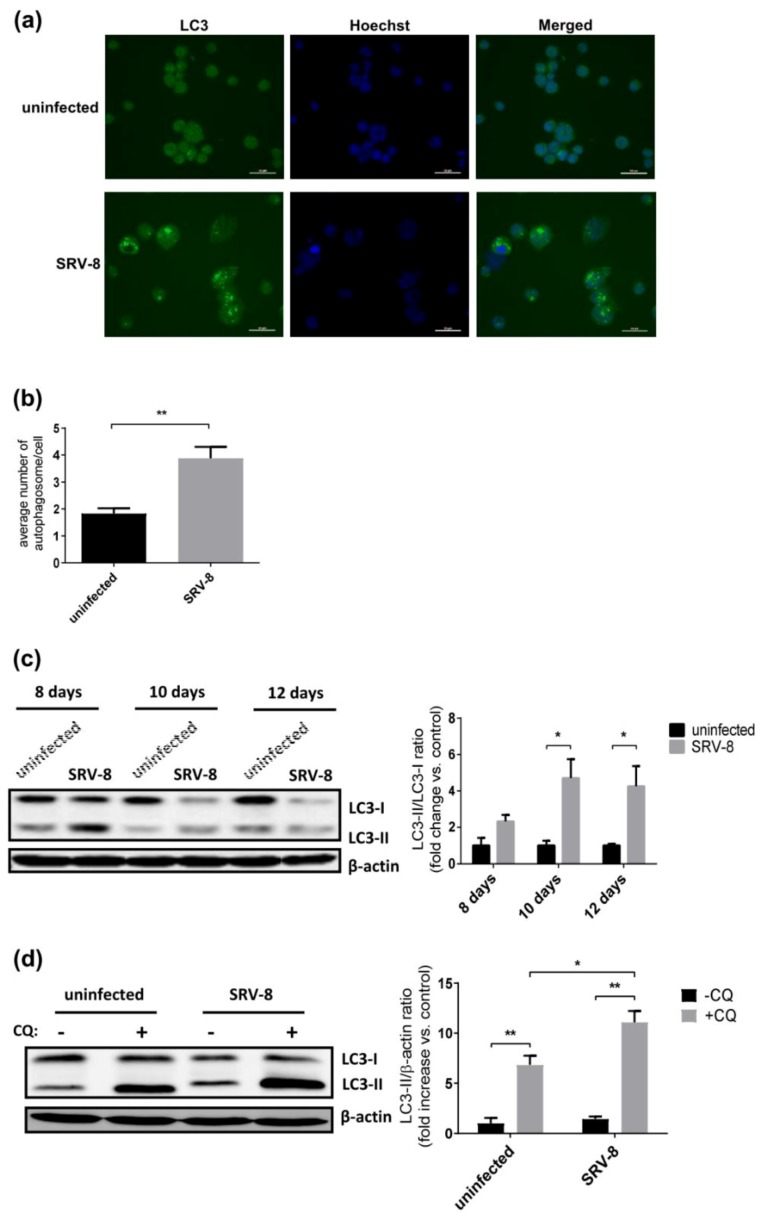
Simian type D retrovirus-8 (SRV-8) infection induces the autophagic flux in Jurkat cells. Jurkat cells were infected with SRV-8, as described in Materials and Methods. (**a**) On day 10 post-infection, uninfected and SRV-8-infected Jurkat cells were stained with anti-LC3 antibody (green) to reveal autophagosomes. Nuclei were visualized by Hoechst staining (blue). Scale bar: 50 µM. (**b**) An average number of LC3 puncta per cell in uninfected and SRV-8-infected Jurkat cells. A minimum number of 100 cells for each condition were analyzed by Image J. (**c**) On day 8, day 10 and day 12 post-infection, lysates of uninfected and SRV-8-infected Jurkat cells were analyzed by western blotting for LC3. β-actin was used as a loading control. The mean folds of LC3-II/LC3-I ratios relative to an uninfected control at each time point were quantified from three independent experiments. (**d**) On day 8 post-infection, uninfected and SRV-8-infected cells were treated with 15 µM chloroquine (CQ) for 48 h. Cell lysates were analyzed by western blotting for LC3. β-actin was used as a loading control. The mean fold changes of LC3-II/β-actin ratios over the basal uninfected and untreated control were quantified from three independent experiments. All of the error bars represent the standard deviation. The data were statistically analyzed using the unpaired Student’s *t*-test (*, *p* < 0.05; **, *p* < 0.01).

Autophagosome formation was quantified in uninfected and SRV-8-infected Jurkat cells by detecting the endogenous LC3 protein, a specific hallmark of autophagosomes, to investigate whether and how the autophagic pathway in Jurkat cells is affected by infection with SRV-8 [48]. Using an immunofluorescent assay, the SRV-8-infected Jurkat cells had an increased number of LC3 puncta on day 10 post-infection relative to uninfected cells (Figure 1a,b), when the viruses were active in replication [47]. This finding suggests that SRV-8 infection increased the accumulation of autophagosomes in Jurkat cells. 

Previous studies have shown that the conversion of soluble LC3-I protein to the lipid bound form, LC3-II, is associated with the formation of autophagosomes [49]. Therefore, the conversion of LC3-I to LC3-II in uninfected and SRV-8-infected cells was examined on day 8, day 10, and day 12 post-infection to investigate whether SRV-8 infection activated the autophagic pathway in Jurkat cells. The ratio of LC3-II/LC3-I on day 10 and day 12 post-infection was significantly increased in infected cells as compared to that in uninfected control cells, suggesting that SRV-8 infection induces autophagosome formation in Jurkat cells (Figure 1c). 

In principle, autophagosome accumulation and an increase in the LC3-II/LC3-I ratio could be due to an increase of autophagosome formation or the inhibition of autophagosome degradation. Therefore, in order to investigate the degree of autophagic flux following SRV-8 infection, the Jurkat cells were treated with chloroquine (CQ), which blocks the fusion of lysosomes with autophagosomes, thereby preventing the degradation of the latter [50]. If SRV-8 infection increased autophagic flux, CQ treatment would lead to an additive increase of LC3-II relative to the untreated cells. On the other hand, if SRV-8 infection blocked the degradation of the autophagosome, no further increase of LC3-II would be observed in response to CQ treatment. On day 8 post-infection, uninfected and SRV-8-infected Jurkat cells were both treated with 15 µM CQ for 48 h. CQ treatment significantly increased the LC3-II level in both uninfected and SRV-8-infected cells as compared to the respective untreated cells, suggesting that SRV-8 infection did not inhibit the degradation of autophagosomes in Jurkat cells (Figure 1d). Moreover, CQ treatment induced a significantly greater amount of LC3-II in the infected cells than in the uninfected cells (Figure 1d), providing additional evidence that the autophagic responses could be enhanced by SRV-8 infection of Jurkat cells. Taken together, the above results indicate that SRV-8 infection enhances autophagic flux in Jurkat cells. 

### 3.2. Inhibition of Autophagy Enhances SRV-8 Replication in Jurkat Cells

**Figure 2:**
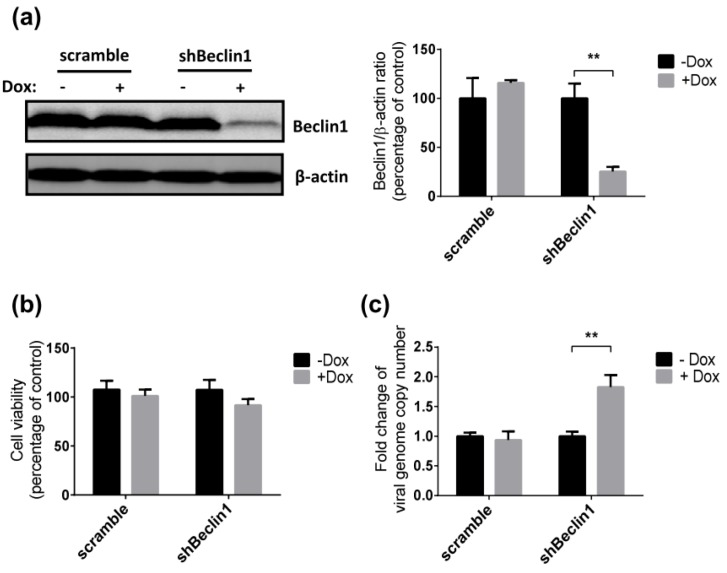
Knockdown of Beclin1 expression enhances SRV-8 replication in Jurkat cells. (**a** and **b**) Dox-inducible scramble and shBeclin1 Jurkat cell lines were constructed by using the lentiviral system. The scramble cell samples were used as the non-silencing control. Scramble and shBeclin1 stable Jurkat cells were induced with 1 µg/mL Dox for three days. (**a**) Lysates of the cells were analyzed by western blotting for Beclin1. β-actin was used as a loading control. The Beclin1/β-actin ratios from three independent experiments were quantified and expressed as the mean percentage of Dox-uninduced control. (**b**) Cell viability of Dox-uninduced (-Dox) and -induced (+Dox) cells were analyzed by MTT colorimetric assay. Data from three independent experiments were quantified and expressed as the percentage of Dox-uninduced control. (**c**) Scramble and shBeclin1 stable Jurkat cells were infected with SRV-8 for six days followed by 1 µg/mL Dox induction for three days. Viral RNA genomes in the culture medium were extracted and the copy numbers were measured by qRT-PCR assay. Data from five independent experiments were quantified and the mean folds relative to Dox-uninduced control are presented. All of the error bars represent the standard deviation. The data were statistically analyzed using the unpaired Student’s *t*-test (**, *p* < 0.01).

Beclin1 is an essential autophagy protein that is known to be important in the initiation of the autophagic pathway. We developed Jurkat cell lines that stably express a doxycycline (Dox)-inducible shRNA targeting Beclin1 (shBeclin1) or a control shRNA (scramble) to investigate the impact of autophagy on SRV-8 replication in Jurkat cells. Efficient downregulation of Beclin1 expression (75%) was observed in Dox-induced shBeclin1 Jurkat cells as compared to uninduced cells (Figure 2a). The cell viability of shBeclin1 Jurkat cells was not affected by Dox-induction (Figure 2b). 

SRV-8 replication was then examined in shBeclin1 and control Jurkat cells. The cells were infected with SRV-8 for six days, followed by Dox induction for 3 days, prior to quantification of viral RNA genomes in the culture media. Dox induction significantly increased the copy numbers of released viral genomes in the culture media of shBeclin1 Jurkat cells. In contrast, there was no significant increase in copy numbers when cells expressing the scrambled shRNA were used (Figure 2c). This finding suggests that SRV-8 replication is enhanced by autophagy deficiency in the Jurkat cells.

### 3.3. SRV-8 Infection Induces Apoptosis and Activation of Caspase-8 in Jurkat Cells

**Figure 3:**
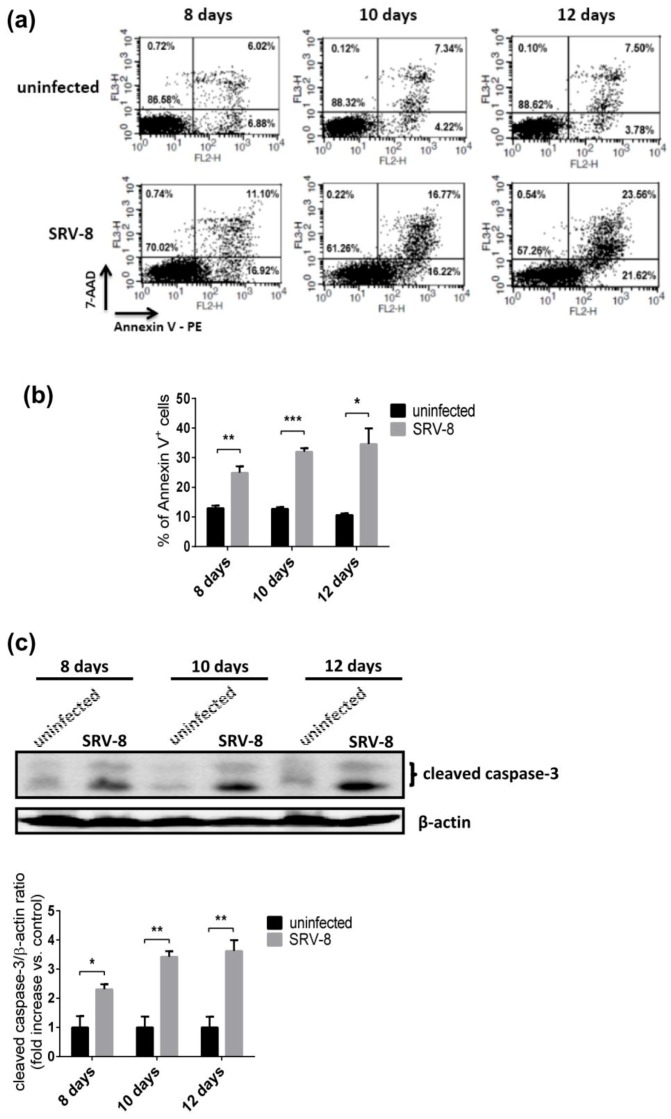
SRV-8 infection induces apoptosis in Jurkat cells. Jurkat cells were infected with SRV-8 as described in Materials and Methods. (**a**) On day 8, day 10, and day 12 post-infection, the apoptosis levels of uninfected and SRV-8-infected Jurkat cells were analyzed using the Annexin V-PE/7-AAD staining assay in conjunction with flow cytometry. The values represent the percentage of cells in each region in one representative experiment. (**b**) Percentage of Annexin V^+^ cells in each sample was quantified from three independent experiments and the mean value is presented. Annexin V^+^ cells include the cells in early (Annexin V^+^/7-AAD^−^) and late (Annexin V^+^/7-AAD^+^) apoptosis. (**c**) On day 8, day 10, and day 12 post-infection, lysates of uninfected and SRV-8-infected Jurkat cells were analyzed by western blotting for cleaved caspase-3. β-actin was used as a loading control. The mean folds of cleaved caspase-3/β-actin ratios relative to an uninfected control at each time point were quantified from three independent experiments. All of the error bars represent the standard deviation. The data were statistically analyzed using the unpaired Student’s *t*-test (*, *p*<0.05; **, *p* < 0.01; ***, *p* < 0.001).

The level of apoptosis in Jurkat cells was firstly examined by flow cytometry following staining with antibodies specific for Annexin V-PE/7-AAD to investigate whether enhanced autophagy affects the apoptotic pathway in SRV-8-infected Jurkat cells. Apoptosis was enhanced in Jurkat cells infected with SRV-8 (Figure 3a). Following infection, the percentage of Annexin V^+^ cells increased to 24.8% on day 8, 32% on day 10, and 34.6% on day 12 post-infection, which were all significantly higher than the values in the uninfected control cells (Figure 3b). Apoptosis is mainly initiated and mediated by cascades of apoptotic caspases, of which caspase-3 is a major executioner caspase. Therefore, we next examined the effect of SRV-8 infection on the activation of caspase-3 in Jurkat cells. The level of cleaved caspase-3 significantly increased in SRV-8-infected Jurkat cells from day 8 to day 12 post-infection relative to its level in uninfected control cells, as shown in Figure 3c. 

**Figure 4:**
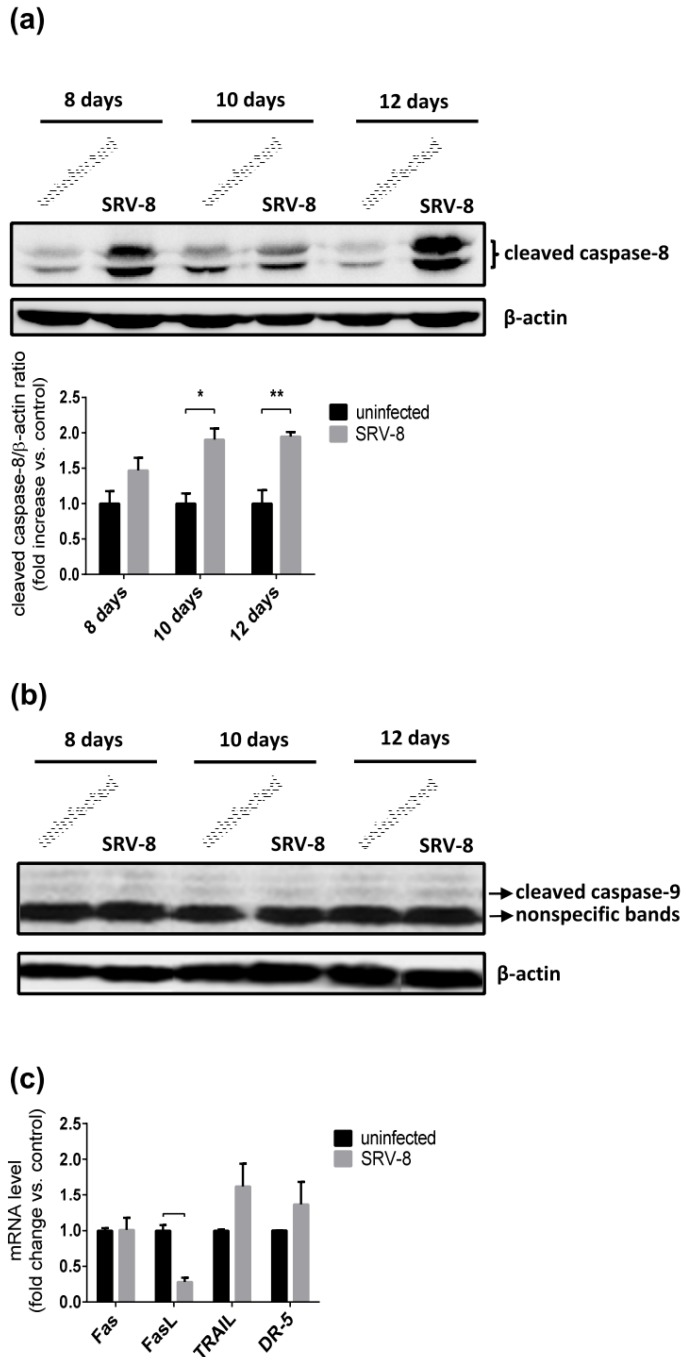
SRV-8 infection enhances activation of caspase-8, but not caspase-9, in Jurkat cells. Jurkat cells were infected with SRV-8 as described in Materials and Methods. (**a** and **b**) On day 8, day 10 and day 12 post-infection, lysates of uninfected and SRV-8-infected Jurkat cells were analyzed by western blotting for (**a**) cleaved caspase-8 and (**b**) cleaved caspase-9. β-actin was used as a loading control. (**a**) The mean folds of cleaved caspase-8/β-actin ratios relative to an uninfected control at each time point were quantified from three independent experiments. (**b**) Representative blots are shown from three independent experiments. Two arrows indicate the nonspecific bands and the expected bands of cleaved caspase-9 protein, respectively. (**c**) On day 10 post-infection, mRNA levels of Fas, FasL, TRAIL, and DR-5 in uninfected and SRV-8-infected Jurkat cells were analyzed by qRT-PCR. GAPDH was used as an internal control. The mean fold changes of the indicated mRNAs relative to an uninfected control were quantified from three independent experiments. All error bars represent the standard deviation. The data were statistically analyzed using the unpaired Student’s *t*-test (*, *p* < 0.05; **, *p* < 0.01; ***, *p* < 0.001).

Because the activation of caspase-3 could result from both intrinsic and extrinsic apoptotic pathways, we then investigated which of the two pathways contribute to SRV-8-induced caspase-3 activation and apoptosis in Jurkat cells. Caspase-9 and caspase-8 are major initiator caspases of the intrinsic and extrinsic apoptotic pathway, respectively. The level of cleaved caspase-8 increased in SRV-8-infected Jurkat cells when compared with the uninfected control cells (Figure 4a). However, the level of cleaved caspase-9 was unchanged in cells that were infected with SRV-8 (Figure 4b). Notably, the increase in the proportion of Annexin V^+^ cells and the levels of cleaved caspase-3/-8 were correlated in SRV-8-infected Jurkat cells, which suggested that SRV-8 infection-induced apoptosis in Jurkat cells resulted from the activation of caspase-8 and caspase-3. 

The levels of mRNAs for Fas/FasL and DR5/TRAIL were measured to determine whether death receptors and their ligands are involved in the activation of caspase-8 in SRV-8-infected Jurkat cells. The mRNA levels of Fas, DR5, and TRAIL did not differ in SRV-8-infected Jurkat cells on day 10 post-infection when caspase-8, caspase-3, and apoptosis were activated in the infected cells when compared with the uninfected control cells (Figure 4c). Indeed, the mRNA level of FasL significantly decreased (to 28%) in the infected cells. These findings suggest that the activation of caspase-8 in SRV-8-infected cells might be independent of death receptors. 

### 3.4. Apoptosis in SRV-8-Infected Jurkat Cells is Regulated by Autophagosome Formation

**Figure 5:**
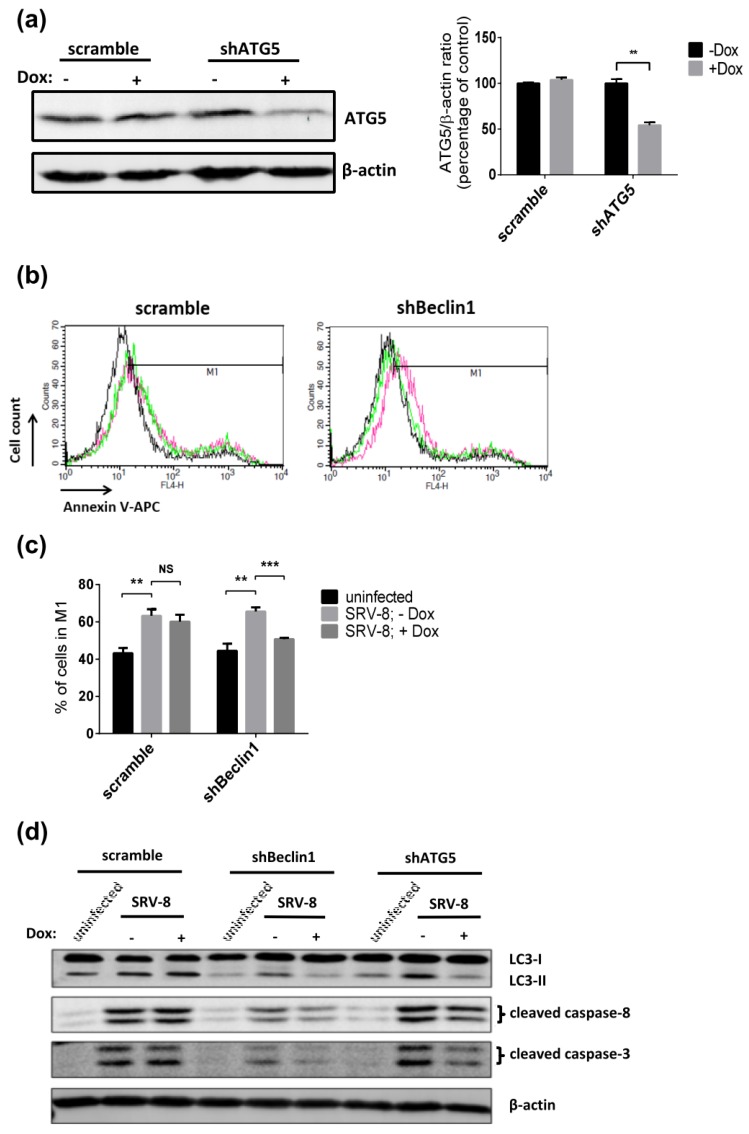

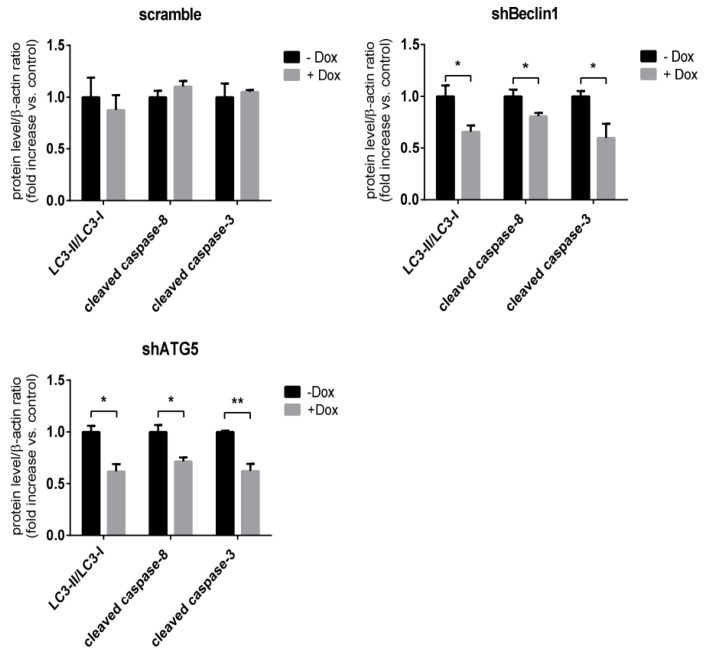
Knockdown of Beclin1 or ATG5 expression reduces SRV-8-induced apoptosis in Jurkat cells. (**a**) Dox-inducible shATG5 Jurkat cell line was constructed by using the lentiviral system. Scramble and shATG5 stable Jurkat cells were induced with 1 µg/mL Dox for 3 days. Lysates of the cells were analyzed by western blotting for ATG5. β-actin was used as a loading control. The ATG5/β-actin ratios from three independent experiments were quantified and expressed as the mean percentage of Dox-uninduced control. (**b**–**d**) Scramble, shBeclin1 and shATG5 stable Jurkat cells were infected with SRV-8 for five days followed by 1 µg/mL Dox induction for 3 days. (**b**) Level of apoptosis of scramble and shBeclin1 Jurkat cells was examined by flow cytometry with Annexin V-APC staining. Each graph includes the data of uninfected cells without Dox induction (black), and SRV-8-infected cells without Dox induction (red) or with Dox induction (green). M1 gates were set to indicate the cell populations with Annexin V^+^ cells. (**c**) Percentage of the cells in M1 gate was quantified from five independent experiments and the mean value is presented. (**d**) The protein levels of LC3, cleaved caspase-8, and caspase-3 were examined by western blot analysis. β-actin was used as a loading control. Data from four independent experiments were quantified and the mean folds of the indicated proteins relative to SRV-8-infected scramble, shBeclin1 or shATG5 Jurkat cells without Dox induction are presented. All of the error bars represent the standard deviation. The data were statistically analyzed using the unpaired Student’s *t*-test (NS: non significant; *, *p* < 0.05; **, *p* < 0.01; ***, *p* < 0.001).

The results described above suggest that autophagy and apoptosis occurred simultaneously in SRV-8-infected Jurkat cells. Accordingly, we next examined whether autophagy that was induced by SRV-8 infection was able to mediate apoptosis in Jurkat cells. Jurkat cells expressing shBeclin1 RNA and scrambled shRNA were infected with SRV-8 for five days, followed by Dox treatment for three days. Apoptosis in the cells was then examined using the Annexin V assay. The knockdown of Beclin1 in the infected cells significantly decreased the percentage of Annexin V^+^ cells, which suggested that the inhibition of autophagy by Beclin1 knockdown was able to rescue SRV-8-induced apoptosis in Jurkat cells (Figure 5b,c). In keeping with this finding, Beclin1 knockdown also significantly decreased the levels of cleaved caspase-8 and caspase-3 in SRV-8-infected shBeclin1 Jurkat cells when compared to that in the Dox-uninduced cells (Figure 5d). Moreover, the knockdown of ATG5, another autophagosomal protein that is a major regulator of autophagy, also suppressed the activation of caspase-8/-3 in SRV-8-infected cells (Figure 5a,d). Additionally, the ratio of LC3-II/LC3-I was significantly reduced in Beclin1 or ATG5 knockdown Jurkat cells, which indicated the successful inhibition of autophagosome formation (Figure 5d). Collectively, our findings suggest that autophagosome formation is involved in the activation of caspase-8/-3 and apoptosis in SRV-8-infected Jurkat cells.

**Figure 6:**
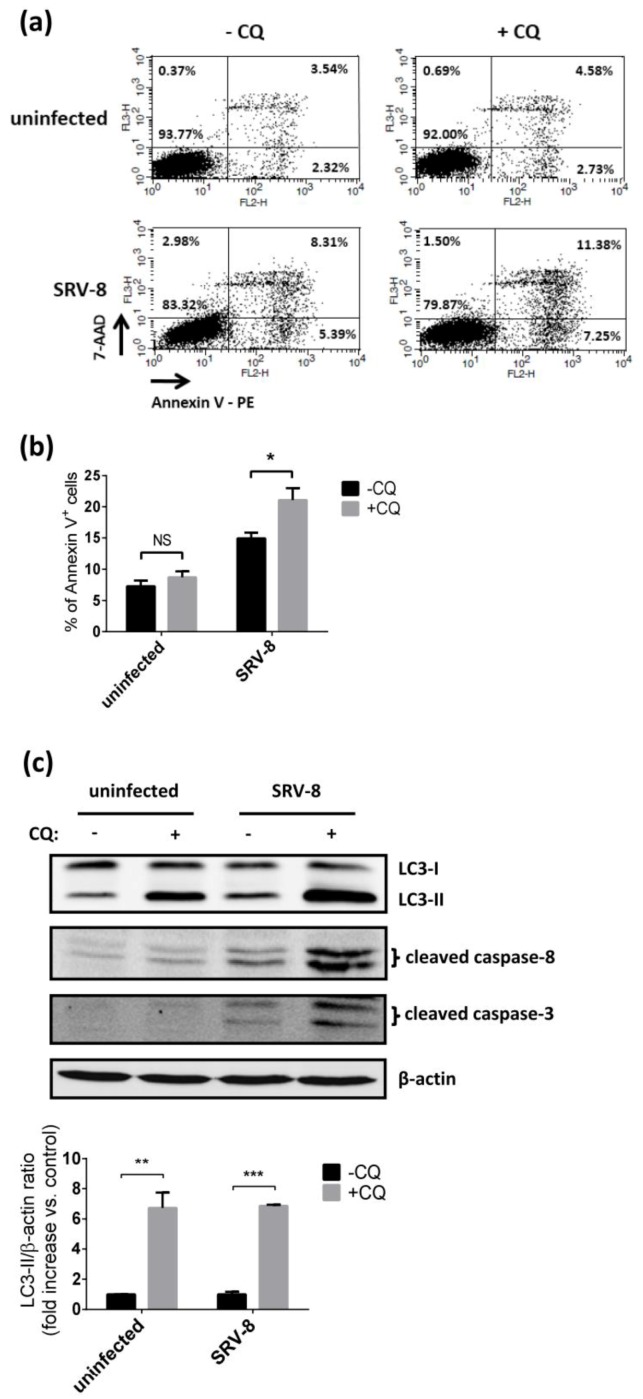

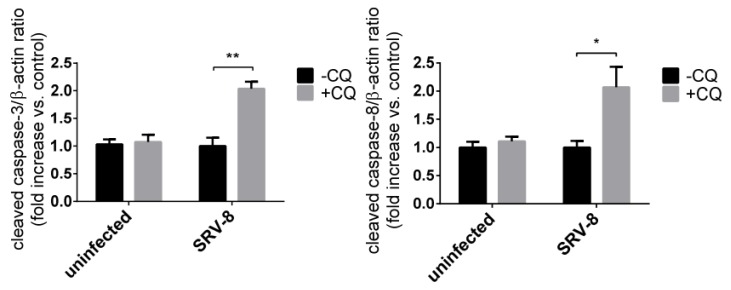
Chloroquine treatment enhances apoptosis in SRV-8-infected Jurkat cells. Jurkat cells were infected with SRV-8 for eight days followed by incubation with 15 µM CQ for 48 h. (**a**) The level of apoptosis was examined by flow cytometry with Annexin V-PE/7-AAD staining. The values represent the percentage of cells in each region in one representative experiment. (**b**) Percentage of Annexin V^+^ cells in each sample was quantified from three independent experiments and the mean value is presented. (**c**) The protein levels of LC3, cleaved caspase-8, and caspase-3 were examined by western blot analysis. β-actin was used as a loading control. Data from five independent experiments were quantified and the mean folds relative to the CQ untreated group for the level of indicated protein are presented. All error bars represent the standard deviation. The data were statistically analyzed using the unpaired Student’s *t*-test (NS: non-signification; *, *p* < 0.05; **, *p* < 0.01; ***, *p* < 0.001).

We used CQ to inhibit the degradation of autophagosome and to examine apoptosis in the infected cells to further understand the functional relevance of autophagosome formation and apoptosis in Jurkat cells during SRV-8 infection. 15 µM CQ was used to treat uninfected and SRV-8-infected Jurkat cells on day 8 post-infection for 48 h. For both uninfected and SRV-8-infected cells, the levels of LC3-II in CQ treated groups were significantly increased relative to the levels in untreated groups, which indicated that degradation of the autophagosome was inhibited, as shown in Figure 6c. Interestingly, CQ treatment could increase the percentage of Annexin V^+^ cells in the SRV-8-infected groups from 14.9% to 21.0%; whereas, the treatment had no effect on the uninfected cells (Figure 6a,b). Furthermore, CQ treatment significantly increased the cleavage of both caspase-8 and caspase-3 in SRV-8-infected cells (Figure 6c). This suggests that CQ treatment enhances apoptosis in Jurkat cells that are infected with SRV-8. 

These results indicate that the inhibition of autophagosome formation by knockdown of Beclin1 or ATG5 suppressed apoptosis in SRV-8-infected Jurkat cells. In contrast, the inhibition of autophagosome degradation by CQ treatment enhanced apoptosis. The findings further suggest that the number of autophagosomes is positively correlated with the degree of apoptosis in SRV-8-infected Jurkat cells. 

### 3.5. Procaspase-8 Localizes to Autophagosomes and Interacts with LC3 and p62/SQSTM1 in SRV-8-Infected Jurkat Cells 

**Figure 7:**
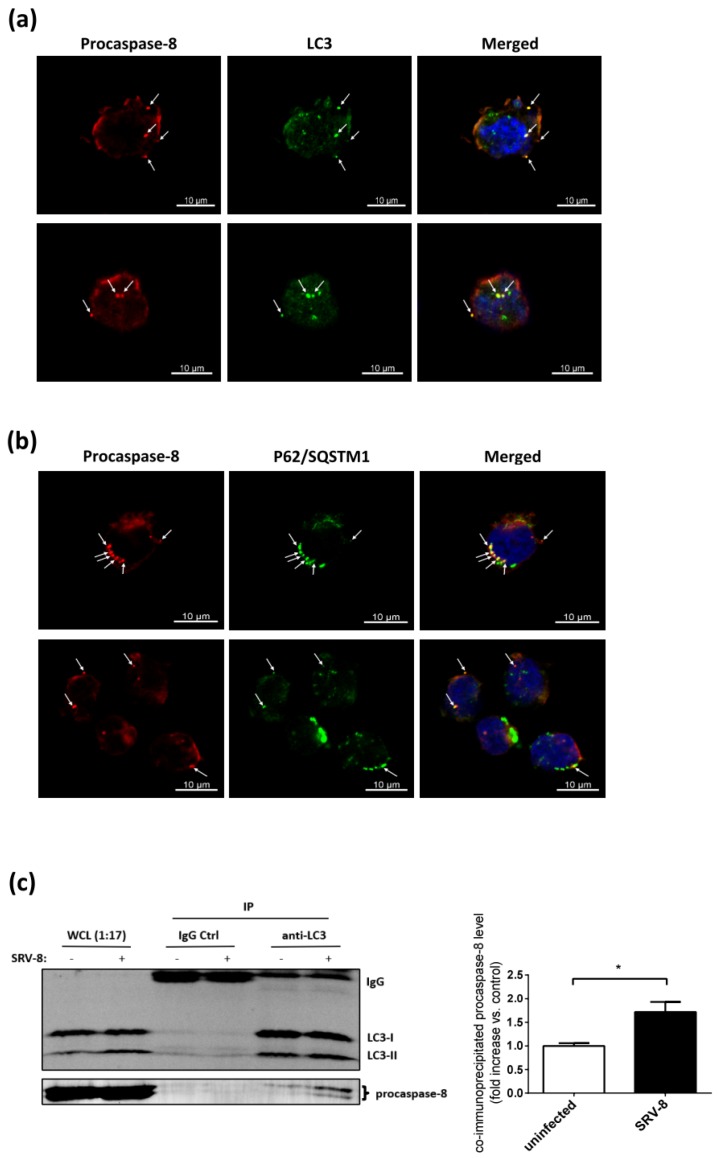
Procaspase-8 localizes on autophagosome, interacts with LC3 and p62/SQSTM1 in SRV-8-infected Jurkat cells. Jurkat cells were infected with SRV-8 as described in Materials and Methods. (a and b) On day 10 post-infection, SRV-8-infected Jurkat cells were doubly stained with (**a**) anti-procaspase-8 (red) and anti-LC3 (green) antibodies or (**b**) anti-procaspase-8 (red) and anti-p62/SQSTM1 (green) antibodies. The white arrows indicate the co-localization as yellow puncta. Nuclei were visualized by Hoechst staining (blue). Scale bar: 10 µM. (**c**) On day 10 post-infection, the cell lysates of uninfected and SRV-8-infected Jurkat cells were subjected to immunoprecipitation with anti-LC3 antibodies or the control rabbit IgG. The levels of LC3 and procaspase-8 in the whole cell lysates (WCL) and the immunoprecipitated (IP) samples were examined using western blot analysis. IgG light chain was served as a loading control of IP samples. Data from four independent experiments were quantified and the mean fold change relative to uninfected cells for the level of co-immunoprecipitated procaspase-8 is presented. All of the error bars represent the standard deviation. The data were statistically analyzed using the unpaired Student’s *t*-test (*, *p* < 0.05).

We investigated whether the formation of autophagosomes in SRV-8-infected Jurkat cells recruited procaspase-8 and mediated its activation, leading to apoptosis in the infected cells, in order to investigate how autophagosomes are involved in apoptosis. Autophagic proteins LC3 and p62/SASTM1, two important markers of autophagosome in mammalian cells, have been shown to be involved in the formation of iDISC and promote the aggregation and self-processing of procaspase-8 [30,32]. Therefore, the co-localization of procaspase-8 with LC3 and with p62/SQSTM1 in SRV-8-infected Jurkat cells was examined using a double immunostaining assay. Several endogenous procaspase-8 puncta were detected in Jurkat cells that were infected with SRV-8 on day 10 post-infection, and a subset of them was co-localized with LC3 puncta (Figure 7a) and with p62/SQSTM1 (Figure 7b). Further evidence for interaction between procaspase-8 and LC3 in SRV-8-infected cells was obtained using a co-immunoprecipitation assay (Figure 7c). An increased amount of procaspase-8 was co-immunoprecipitated with LC3 in the infected cells relative to the uninfected control cells. These findings suggest that the formation of autophagosomes is at least partially involved in the process of caspase-8 activation in SRV-8-infected Jurkat cells. 

## 4. Discussion

SRV infection can cause lymphocyte depletion and immunosuppression in macaques, which are similar to the acquired immunodeficiency syndrome (AIDS) found in human individuals that are infected with human immunodeficiency virus (HIV) [51]. However, the mechanism of lymphoid cell depletion induced by SRV is still unclear. Previous studies have shown that HIV-induced autophagy is involved in T cell death, which, in turn, is likely to contribute to immunodeficiency [52,53,54]. Autophagy has also been recognized as an evolutionarily conserved host immune defense mechanism that plays an essential role against viral infection [12,13]. Nevertheless, some viruses have developed strategies for suppressing the autophagic pathway for their own benefits [4,14,15,16]. To date, it remains unclear whether SRV infection interferes with autophagy in T lymphocytes. Unlike HIV, which has a narrow cell tropism, SRV has a broad cell tropism for lymphoid and nonlymphoid cell types in vivo [39] and it can also infect several human T- and B-cell lines in vitro [45,46,55]. Meanwhile, Jurkat T cells have been commonly used as an in vitro model to study the interactions between viruses and T cells [8,56]. Here, we used SRV-8-infected human Jurkat cells as a model system to examine the role of autophagosome and apoptosis in virus-induced death of T lymphocytes. SRV-8 is a new subtype that was recently discovered from cynomolgus monkeys [43]. Phylogenetic analysis and serological examination have shown [43] that SRV-8 is more closely related to SRV-4, which can cause a lethal hemorrhagic syndrome when transmitted to Japanese macaques [57]. We have previously reported evidence showing that the expression of SRV proviral long terminal repeat (LTR) inside Jurkat cells and viral genome copies that are released into the culture medium gradually increased from two days to 10 days post-infection and tended to stabilize thereafter [47]. The productive infection of Jurkat cells with SRV-8 resembles SRV-1 infection of other human T cell lines, where the envelope gp20 protein is readily expressed after 10 days infection [45]. Thus, SRV-8 infection of Jurkat cells provides an in vitro model for analysis of interactions between SRV and T cells. 

The antiviral function of autophagy has been extensively reported in the previous studies [4,58]. As an essential cellular mechanism for degrading the unwanted cytoplasmic components, the autophagic process has been shown to function as an intrinsic cellular defense mechanism to eliminate invading viruses and to inhibit viral replication [4,10,11,59,60,61]. Previous studies have demonstrated that the replication of Sindbis virus and herpes simplex virus type 1 was reduced by the upregulation of autophagy in infected cells [62,63]. The inhibition of viral replication by autophagy has also been reported for Japanese encephalitis virus, which is a positive strand RNA virus; and, the decreased expression of autophagy-related genes (ATG), such as ATG5 or ATG7, was shown to enhance the viral replication [11]. In agreement with the above studies, our results demonstrated that autophagic flux in Jurkat cells was enhanced by SRV-8 infection and inhibition of autophagy, achieved by knocking down of Beclin1 expression, enhanced SRV-8 replication in the Jurkat cells. These findings support the idea that autophagy functions as a restriction mechanism against SRV-8 replication in Jurkat cells. However, further studies are warranted to decipher the precise molecular mechanism(s) of the autophagy-mediated anti-SRV effect, with a particular emphasis on the identification of autophagic protein(s) that functions in targeting SRV-8 virions in Jurkat cells. It would also be interesting to understand how SRV-8 counteracts the antiviral action of autophagy in order to favor its replication in Jurkat cells. 

As a type II programmed cell death signal, autophagy can operate independently or cooperatively with apoptosis to determine the fate of cells [27,64,65,66]. Here, we first observed an increased ratio of Annexin V^+^ cells while using flow cytometry when the Jurkat cells were infected with SRV-8. Furthermore, we demonstrated that both caspase-3 and caspase-8, but not caspase-9, were activated during SRV-8 infection of Jurkat cells. These results suggest that SRV-8 infection might enhance apoptosis of Jurkat cells through the activation of an extrinsic apoptosis pathway. Nevertheless, the expression of death receptors and their ligands, the direct activators of caspase-8, was not affected by SRV-8 infection. In addition, apoptosis that was induced by SRV-8 infection closely coincided temporally with an accumulation of autophagosomes. Of relevance to this effect, we found that, in SRV-8 infected Jurkat cells, the inhibition of autophagosome formation, as mediated by the knockdown of Beclin1 or ATG5, resulted in a marked suppression of caspase-8/-3 activation and apoptosis. These results corroborate several studies indicating that autophagy is involved in the apoptosis of T lymphocytes during viral infection [25,53]. It also should be noted that the autophagosome formation is sufficient for triggering the apoptosis of Jurkat cells during SRV infection. In our study, Chloroquine (CQ), which is known to block the fusion of autophagosomes with lysosomes and, therefore, to prevent the degradation of autophagosomes, could enhance caspase-8/-3 activation and the apoptosis of SRV-8 infected Jurkat cells.

Our study further demonstrates that procaspase-8 co-localizes with LC3 and p62/SQSTM1 on the autophagosomes and the increased interaction between procaspase-8 and LC3 in the Jurkat cells infected with SRV-8. Therefore, these observations indicate that the induction of autophagy that is triggered by SRV-8 infection plays an important role in recruiting procaspase-8 to the expanding autophagosomal membrane to initiate caspase-8/-3 cascade in the Jurkat cells, which are in line with previous reports showing that the autophagosomal membrane serves as a platform for the formation of iDISC to mediate the activation of caspase-8 [30,32,67,68,69]. So far, several autophagic proteins have been proposed as the components of iDISC, including LC3, p62/SQSTM1, and ATG5 [32,68,70,71]. We also noticed that the interaction between LC3 and procaspase-8 was enhanced during SRV-8 infection of Jurkat cells. However, whether and how autophagic protein(s) participates in the formation of iDISC for the activation of caspase-8 in the infected Jurkat cells remains under further investigation. 

In conclusion, this study reveals for the first time that both autophagic and apoptotic pathways are enhanced in the SRV8-infected Jurkat cell model system, and that the induced autophagy might function as a defense mechanism to inhibit virus replication and induce cell death through apoptosis. Moreover, we provide the first evidence that enhanced autophagosome formation following SRV-8 infection recruits procaspase-8 for its activation, which then might induce the downstream caspase cascade and lead to the apoptosis of infected Jurkat cells. Our findings may provide a new approach for understanding the loss of T lymphocyte and immunosuppression during SRV infection.
